# Coronary artery calcium, HIV and inflammation in Uganda compared with the USA

**DOI:** 10.1136/openhrt-2019-001046

**Published:** 2019-05-22

**Authors:** Ben Alencherry, Geoffrey Erem, Grace Mirembe, Isaac Ssinabulya, Chun-Ho Yun, Chung-Lieh Hung, Mark J Siedner, Marcio Bittencourt, Cissy Kityo, Grace A McComsey, Chris T Longenecker

**Affiliations:** 1 Medicine and Pediatrics, University Hospitals Cleveland Medical Center, Cleveland, Ohio, USA; 2 Radiology, St Francis Hospital Nsambya, Kampala, Uganda; 3 Radiology and Medicine, Makerere University College of Health Sciences, Kampala, Uganda; 4 HIV Medicine, Joint Clinical Research Centre, Kampala, Uganda; 5 Cardiology, Uganda Heart Institute, Kampala, Uganda; 6 Radiology, Mackay Memorial Hospital, Taipei, Taiwan; 7 Cardiology, Mackay Memorial Hospital, Taipei, Taiwan; 8 Infectious Diseases, Massachusetts General Hospital, Boston, Massachusetts, USA; 9 Medicine, Harvard Medical School, Boston, Massachusetts, USA; 10 Division of Internal Medicine, University Hospital, Sao Paulo, Brazil; 11 Pediatric Infectious Diseases, Case Western Reserve University School of Medicine, Cleveland, Ohio, USA; 12 Cardiology, Case Western Reserve University School of Medicine, Cleveland, Ohio, USA

**Keywords:** HIV, coronary artery calcium, sub-saharan africa, inflammation

## Abstract

**Objectives:**

To compare the prevalence of detectable coronary artery calcium (CAC) among higher risk, older people living with HIV (PLWH) and uninfected persons in Uganda versus the USA, and second to explore associations of CAC with HIV-specific variables and biomarkers of inflammation.

**Methods:**

This cross-sectional study of 430 total subjects compared 100 PLWH on antiretroviral therapy and 100 age-matched and sex-matched HIV-uninfected controls in Uganda with 167 PLWH on antiretroviral therapy and 63 uninfected controls in the USA. Multivariable logistic regression was used to examine associations with detectable CAC (CAC >0).

**Results:**

Compared with US subjects, Ugandans were older (mean age 56 vs 52 years) and were more likely to have diabetes (36% vs 3%) and hypertension (85% vs 36%), but were less likely to be male (38% vs 74%) or smokers (4% vs 56%). After adjustment for HIV serostatus, age, sex and traditional risk factors, Ugandans had substantially lower odds of CAC >0 (adjusted OR 0.07 (95% CI 0.03 to 0.17), p<0.001). HIV was not associated with CAC >0 in either country (p>0.1). Among all PLWH, nadir CD4 count was associated with the presence of CAC, and among Ugandans soluble intercellular adhesion molecule (p=0.044), soluble CD163 (p=0.004) and oxidised low-density lipoprotein (p=0.043) were all associated with the presence of CAC.

**Conclusions:**

Ugandans had a dramatically lower prevalence of any coronary calcification compared with US subjects. The role of HIV infection and inflammation as risk factors for subclinical coronary disease in sub-Saharan Africa merits further investigation.

**Key questions**

**What is already known about this subject?**

- Ischaemic heart disease may be on the rise in sub-Saharan Africa due to recent increases in the prevalence of traditional risk factors and non-traditional risk factors such as HIV infection.
- In addition, modelling analyses using Global Burden of Disease (GBD) Study data suggest that HIV may contribute up to 10%–15% of the population attributable risk for atherosclerotic cardiovascular disease events in sub-Saharan Africa; however, these studies rely on assumptions about risk and disease burden that incorporate very few primary data from the region.

**What does this study add?**

- This study suggests that despite high rates of risk factors, the prevalence of detectable coronary calcium is much lower among Ugandans compared with US subjects.
- One implication of these findings is that the risk of atherosclerotic coronary heart disease events may be lower in Uganda than predicted by the GBD.
- Finally, our findings suggest that inflammation and immune activation may be important risk factors for coronary artery disease in sub-Saharan Africa, particularly for people living with HIV.

**How might this impact on clinical practice?**

- These estimates of subclinical cardiovascular disease burden will inform future research on clinical outcomes and may inform health policymakers in low-income countries concerned about controlling non-communicable diseases.
- Our findings should prompt additional investigation of the role of traditional and non-traditional risk factors (such as HIV and inflammation) in the development of coronary disease and clinical cardiovascular events in sub-Saharan Africa.

## Introduction

In many parts of sub-Saharan Africa (SSA), the prevalence of cardiovascular disease (CVD) appears to be increasing due to rising rates of traditional atherosclerotic cardiovascular disease (ASCVD) risk factors such as obesity, diabetes and hypertension^1^; however, non-traditional risk factors such as chronic infections and exposure to indoor air pollution may also play a role. For example, chronic HIV infection has been associated with a twofold higher risk of ASCVD and is highly prevalent in SSA.^2^ Modelling studies suggest that HIV may contribute up to 10%–15% of the population attributable risk for ASCVD events in the region.^2^ Yet, although risk factors point to increasing risk, little is known about the true prevalence of subclinical vascular disease or clinical ASCVD risk among people living with HIV (PLWH) in SSA.^3^ Some studies of carotid intima-media thickness^4^ and various measures of arterial stiffness^5–7^ have come to mixed conclusions on the relationship between HIV and subclinical vascular disease in varied SSA populations. Yet no prior studies have directly measured the prevalence of subclinical coronary disease among PLWH and controls in SSA using coronary artery calcium (CAC) scores.

The inflammatory milieu of chronic HIV infection that persists despite antiretroviral therapy (ART)—reflected by increased biomarkers of endothelial and monocyte activation in peripheral blood—may explain the higher ASCVD risk among PLWH.^8^ Studies of inflammation biomarkers and subclinical vascular disease among PLWH have, however, been mixed,^9^ with markers of monocyte activation (eg, soluble CD163 (sCD163) and soluble CD14 (sCD14)) showing the most consistent positive associations with the disease.^10–12^ In rural south-west Uganda, higher sCD14 and interleukin-6 (IL-6) levels 6 months after ART initiation appear to be associated with higher carotid intima-media thickness years later.^13^ However, few other studies in the region have examined the association between inflammation and subclinical vascular disease among PLWH in SSA, and none have done so using CAC as a measure of coronary atherosclerosis.

Therefore, we sought to describe the prevalence of detectable CAC among higher risk, older Ugandans with and without HIV in comparison with persons with and without HIV in Cleveland, Ohio, USA. As a secondary objective, we explored associations between calcified coronary plaque and HIV-specific factors in Uganda, including biomarkers of inflammation and immune activation.

## Methods

### Participant selection

From April 2015 to May 2017, 100 PLWH on ART over 45 years of age and in care at Joint Clinical Research Centre HIV clinic near Kampala were enrolled in the study. For each PLWH, we prospectively identified 100 age-matched and sex-matched HIV-uninfected control participants recruited from the community or hospital-based internal medicine clinics. All had ≥1 major cardiovascular risk factor (hypertension, diabetes mellitus, smoking or high cholesterol). Next we identified 167 PLWH and 63 uninfected controls older than 40 years of age from Cleveland, USA included in a clinical research registry of studies conducted between 2012 and 2017 by the same principal investigators (GAM and CTL).

### Study procedures

For both cohorts, self-reported demographics, smoking status, and medical history including HIV history and hepatitis B/C status were obtained using standardised questionnaires and clinical chart review. Diabetes and hypertension were defined as having a self-reported history of the condition or being on a medication for the condition. Among PLWH, we also recorded current and nadir CD4+ count, time since HIV diagnosis, current ART and total duration of ART. A physical examination was conducted that included height, weight, waist and hip measurements, and blood pressure. All HIV-uninfected subjects were confirmed to be negative with a rapid HIV test. After a 12-hour fast, blood was drawn for clinical labs, including a lipoprotein panel and kidney function. Plasma was stored at −80°C for batched measurements of biomarkers. In Cleveland, HIV-1 RNA level was obtained as part of routine clinical care, but at the time of this study it was not yet available as part of routine care in Uganda.

All subjects underwent non-contrast, ECG-gated cardiac CT for calcium scoring. Scans were performed on a 128-slice multidetector CT scanner in Uganda (Siemens; Munich, Germany) and 64-slice scanner in Cleveland (Siemens). Tube voltage (120–130 kV), gating interval (60%–70% of the RR) and slice thickness (2.5–3 mm) were similar in Uganda and Cleveland. CAC score was measured offline by local radiologists using similar Siemens Syngo and Leonardo workstations. Calcified lesions were defined as having ≥6 pixels with density >130 Hounsfield units, and total CAC score was calculated using the Agatston method.^14^

Biomarkers of inflammation and immune activation were measured in batch from cryopreserved plasma samples collected from Ugandan subjects only. IL-6 was measured by electrochemiluminescence (Meso Scale Diagnostics, Rockville, Maryland, USA). Soluble vascular cell adhesion molecule (R&D Systems, Minneapolis, Minnesota, USA), soluble tumour necrosis factor α receptor II (R&D Systems), soluble intercellular adhesion molecule (sICAM; R&D Systems), sCD14 (R&D Systems), sCD163 (R&D Systems) and oxidised low-density lipoprotein (oxLDL; ALPCO, Salem, New Hampshire, USA) were measured by ELISA. Fibrinogen and high-sensitivity C reactive protein were measured by nephelometry (Siemens).

### Statistical analysis

We first described the demographics and cardiometabolic risk profile of study participants by study group and HIV status. Statistical comparisons between groups (HIV vs control within country; and Uganda vs US) were made using t-tests and Wilcoxon rank-sum tests for continuous variables, and χ^2^ or Fisher’s exact tests for categorical variables as distributionally appropriate. Missingness was <2% for all variables except current CD4+ T cell count (missing n=31 of 267 total PLWH; 12%).

In the primary analysis, we graphically depicted the prevalence of CAC=0, CAC 1–100 and CAC >100 among the four study subgroups. We used logistic regression to determine correlates of detectable calcified coronary plaque (defined as CAC >0; since numbers in the >100 category were too small). We examined the effect of country and HIV status in an (1) unadjusted model, (2) model adjusted for age and sex, and (3) model fully adjusted for traditional risk factors (HIV status, country, age, sex, diabetes, hypertension, current smoking and total cholesterol). We tested a country × HIV interaction in the final model. In two sensitivity analyses, we separately excluded from the final model subjects of non-black race and smokers. We repeated the fully adjusted multivariable model among Ugandan subjects only to examine the effects of these traditional risk factors in this population.

Finally, we explored associations between CAC >0 and each of the following HIV-specific variables among PLWH in the USA and Uganda: current CD4, nadir CD4, time since HIV diagnosis, ART duration, current abacavir use and current protease inhibitor (PI) use. Similar to the primary analysis, we first built unadjusted models, then models adjusted only for country, and finally demographics adjusted and fully adjusted models. In a similar fashion, we used unadjusted and adjusted logistic regression to explore associations of CAC >0 with each of the individual biomarkers measured in Ugandan subjects only. For the inflammation models, we additionally examined whether associations with inflammation biomarkers varied by HIV status by adding an HIV × biomarker interaction term to the final model. For interactions that were significant at p≤0.1, we obtained adjusted OR (AOR) for the biomarker effect in separate models of PLWH only versus HIV-uninfected controls only.

A flow chart of the statistical analyses (online supplementary file 1) describes which analyses were performed on the combined data set versus the Uganda data set only. STATA V.14.0 was used for analysis; p<0.05 was considered statistically significant.

10.1136/openhrt-2019-001046.supp1 Supplementary data

## Results

The characteristics of the study participants are displayed in table 1. Ugandans were more likely to be older (p<0.001) and female (p<0.001). Compared with HIV-negative Ugandans, PLWH in Ugandans had lower rates of diabetes (p<0.001), higher rates of hypertension (p<0.001) and higher high-density lipoprotein cholesterol (p=0.025). Although PLWH in Uganda had lower body mass index (p=0.009), their waist to hip ratio was higher (p<0.001). Compared with PLWH from the USA, PLWH in Uganda had modestly shorter duration of HIV infection (p<0.001) and modestly lower current CD4 (p=0.014); however, nearly all participants had a current CD4 count >200. ART regimens differed substantially, with more PI and integrase inhibitor use in the USA (p<0.001).

**Table 1:** Baseline characteristics of study participants

	Uganda			Cleveland			P value Uganda vs Cleveland
	HIV-negative n=100	HIV-positive n=100	P value	HIV-negative n=63	HIV-positive n=167	P value	
Demographics							
Age	55 (51–60)	55 (51–60)	0.474	53 (49–57)	50 (46–55)	0.003	<0.001
Male	38%	38%	0.999	62%	78%	0.011	<0.001
African American race	NA	NA	NA	49%	65%	0.032	NA
Cardiometabolic risk factors							
Diabetes	45%	26%	0.005	7.9%	1.2%	0.018	<0.001
Hypertension	80%	89%	0.079	25%	40%	0.038	<0.001
Hepatitis B or C	0%	0%	0.999	1.6%	7.8%	0.120	<0.001
Family history of MI	4%	3%	0.999	43%	33%	0.144	<0.001
Current smoker	4%	4%	0.999	47%	60%	0.082	<0.001
Body mass index	30 (26–34)	27 (23–32)	0.014	28 (25–31)	26 (23–29)	0.191	0.009
Waist to hip ratio	0.88 (0.84–0.94)	0.91 (0.86–0.99)	<0.001	0.97 (0.90–1.04)	0.95 (0.90–1.00)	0.679	<0.001
Systolic BP (mm Hg)	152 (135–175)	152 (140–167)	0.398	126 (118–138)	124 (114–138)	0.320	<0.001
Total cholesterol (mg/dL)	5.5 (4.7–6.3)	5.6 (4.5–6.5)	0.423	4.8 (3.9–5.5)	4.5 (4.0–4.9)	0.048	<0.001
LDL cholesterol (mg/dL)	3.6 (2.9–4.4)	3.5 (2.8–4.3)	0.918	2.8 (2.0–3.4)	2.5 (1.9–2.9)	0.014	<0.001
HDL cholesterol (mg/dL)	1.3 (1.1–1.6)	1.5 (1.2–1.8)	0.012	1.3 (1.0–1.6)	1.2 (1.0–1.5)	0.479	0.025
Statin use	5%	7%	0.767	21%	11%	0.051	<0.001
BP medication use	52%	45%	0.322	25%	38%	0.066	0.004
HIV history							
Time since HIV diagnosis (years)	–	12 (10–13)	–	–	15 (9.3–20)	–	<0.001
Current CD4+ (cells/mm^3^)	–	531 (408–676)	–	–	629 (442–860)	–	0.0145
Nadir CD4+ (cells/mm^3^)	–	141 (68–215)	–	–	152 (63–290)	–	0.111
Viral load <48 copies/mL	–	NA	–	–	81%	–	NA
Duration of ART (years)	–	11 (8.8–12)	–	–	8.8 (4.2–14)	–	0.244
Current protease inhibitor	–	20%	–	–	47%	–	<0.001
Current integrase inhibitor	–	0%	–	–	33%	–	<0.001
Current abacavir		6.0%			13%		0.001

ART, antiretroviral therapy; BP, blood pressure; HDL, high-density lipoprotein; LDL, low-density lipoprotein; MI, myocardial infarction; NA, not applicable.

**Figure 1:**
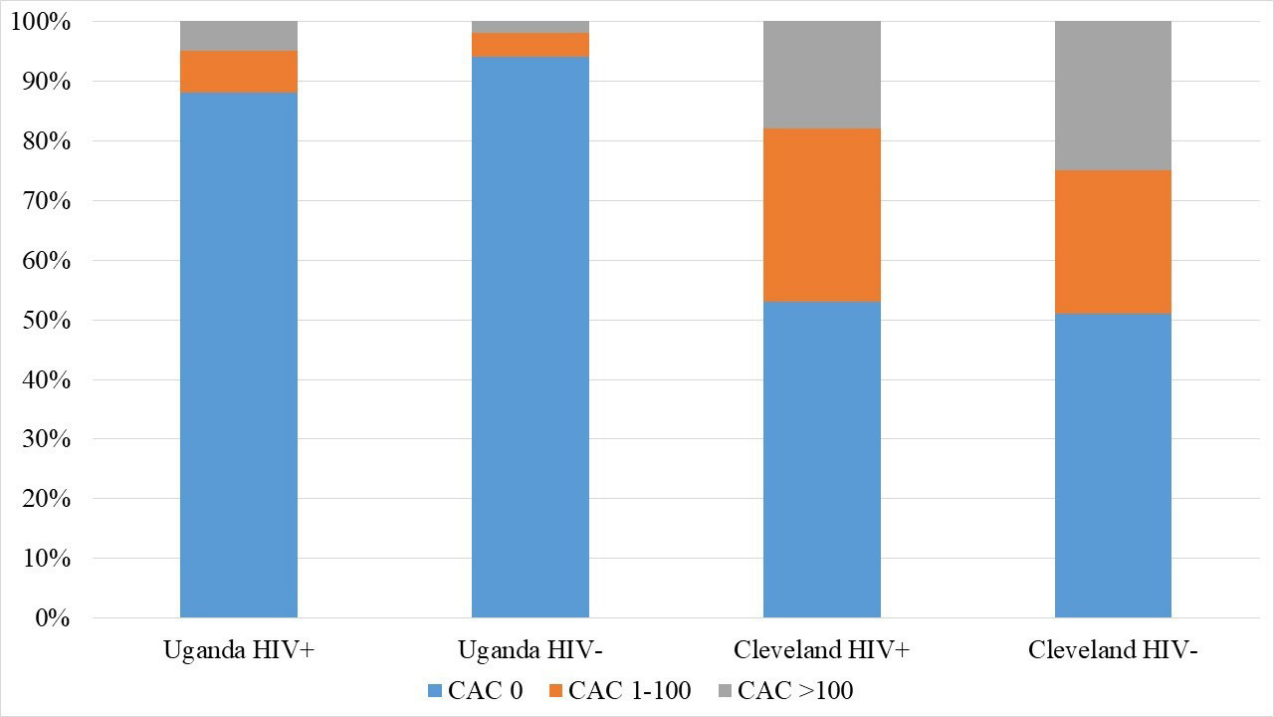
Distribution of coronary artery calcium (CAC) scores among people living with HIV and HIV-uninfected study participants in Uganda and the USA.

Figure 1 shows categorical CAC scores (0, 1–100, >100) by country and HIV status. CAC scores were substantially lower among Ugandans compared with US subjects (p<0.001); in fact, 91% of Ugandans had no detectable coronary calcium. Within-country differences in CAC prevalence between PLWH and HIV-negative participants were not statistically significant (12% vs 6% for PLWH vs HIV-negative in Uganda, p=0.108; 47% vs 49% in the USA, p=0.797); however, when countries were pooled together, the prevalence of CAC >0 was higher among PLWH (34% vs 23%, PLWH vs HIV-negative; p=0.012).

In multivariable logistic regression models, Ugandan subjects were significantly less likely to have CAC >0 (vs US subjects) after adjustment for age, sex and HIV status (AOR 0.07 (95% CI 0.04 to 0.14), p<0.001). The effect remained unchanged after further adjustment for diabetes, hypertension, current smoking and total cholesterol (AOR 0.07 (95% CI 0.03 to 0.17), p<0.001). HIV infection was not associated with CAC >0 in the final model (AOR 1.23 (95% CI 0.70 to 2.18), p=0.470), and there was no statistically significant HIV × country interaction (p=0.20). Excluding smokers (n=128 in the USA and n=8 Uganda) or those of non-black race (n=91 in the USA) did not significantly alter the protective effect of Ugandan nationality (AOR 0.09 (95% CI 0.03 to 0.23) and AOR 0.15 (95% CI 0.06 to 0.39), p<0.001), respectively. When the fully adjusted model was restricted to Ugandan subjects only (table 2), current smoking (p=0.039) and age (p=0.036) were associated with CAC >0. PLWH in Uganda had a non-significant increased odds of CAC >0 (AOR 2.2 (95% CI 0.74 to 6.4), p=0.157).

**Table 2:** Multivariable model of predictors of detectable coronary calcium (CAC >0) among Ugandan participants

	AOR	95% CI	P value
HIV	2.2	0.74 to 6.4	0.157
Age (per decade)	2.3	1.1 to 5.1	0.036
Male sex	1.4	0.49 to 3.9	0.548
Diabetes	1.3	0.41 to 4.4	0.629
Hypertension	5.4	0.50 to 59.2	0.167
Current smoker	10.6	1.1 to 99.3	0.039
Total cholesterol (per mg/dL)	1.0	0.99 to 1.01	0.632

AOR, adjusted OR; CAC, coronary artery calcium.

In models including all PLWH in Uganda and the USA (n=267), the only variable associated with CAC in fully adjusted models was nadir CD4+ count (AOR 0.72 (95% CI 0.56 to 0.94) per unit increase in natural log(ln)-transformed nadir CD4+, p=0.017). Current PI use was associated with CAC in unadjusted models (OR 1.95 (95% CI 1.16 to 3.29), p=0.012), but further adjustment for country, age, sex and traditional risk factors attenuated this relationship (p>0.1).

There were modest differences in biomarkers between HIV-positive and HIV-negative Ugandans. sCD14 was higher in Ugandan PLWH (median (IQR) 1684 (1418–2061) vs 1383 (1156–1601) pg/mL, PLWH vs HIV-negative; p<0.001), whereas fibrinogen (312 (253–378) vs 366 (296–428) mg/dL, PLWH vs HIV-negative; p=0.001) and oxLDL (49 (31–88) vs 70 (49–161), PLWH vs HIV-negative; p<0.001) were lower in Ugandan PLWH. All other biomarkers did not vary by HIV status (p>0.15). The association of inflammation and immune activation markers with coronary calcification in Uganda is shown in table 3. In models that adjusted for HIV status, age, sex and traditional risk factors, concentrations of sICAM, sCD163 and oxLDL were all positively associated with CAC at p<0.05. Furthermore, the strength of the inflammation–CAC relationship appeared to be much stronger among PLWH compared with HIV-negative Ugandans for sCD163 (AOR 25.9 (95% CI 3.46 to 193.3) for PLWH vs 1.79 (95% CI 0.21 to 15.31) for HIV-negative; p for interaction=0.03) and statistically borderline stronger for oxLDL (AOR 2.14 (95% CI 1.17 to 3.94) for PLWH vs 0.87 (95% CI 0.27 to 2.80) for HIV-negative; p for interaction=0.10).

**Table 3:** Association of biomarkers of inflammation and immune activation with detectable coronary calcium (CAC >0) among Ugandan participants (n=200)

	Unadjusted		Multivariable adjusted^*^	
	**OR (95% CI**)	**P value**	**AOR**	**P value**
IL-6	1.20 (0.67 to 2.16)	0.545	1.15 (0.61–2.18)	0.666
sVCAM	2.29 (0.65 to 8.10)	0.199	2.21 (0.53–9.19)	0.277
sTNF-RII	4.81 (0.95 to 24.3)	0.057	4.41 (0.71–27.3)	0.110
hsCRP	0.94 (0.65 to 1.37)	0.748	0.94 (0.63–1.41)	0.780
sICAM	3.06 (0.97 to 9.69)	0.057	4.11 (1.04–16.2)	0.044
Fibrinogen	1.09 (0.28 to 4.34)	0.899	1.13 (0.18–7.15)	0.897
sCD14	3.49 (0.56 to 21.8)	0.182	1.94 (0.26–14.6)	0.520
sCD163	3.77 (1.27 to 11.2)	0.017	6.40 (1.82–22.6)	0.004
oxLDL	1.33 (0.89 to 2.00)	0.161	1.58 (1.01–2.46)	0.043

The unadjusted and multivariable adjusted effect size of the association with CAC >0 is shown separately for each biomarker.

*Adjusted for HIV status, age, sex diabetes, hypertension, current smoking and total cholesterol. All biomarkers were natural log (ln)-transformed prior to analysis. The effect size is the odds ratio for CAC >0 per 1 unit increase in ln-transformed biomarker.

AOR, adjusted OR; CAC, coronary artery calcium; IL-6, interleukin 6; hsCRP, high-sensitivity C reactive protein; oxLDL, oxidised low-density lipoprotein; sCD14, soluble CD14; sCD163, soluble CD163; sTNF-RII, soluble tumour necrosis factor α receptor II; sVCAM, soluble vascular cell adhesion molecule; siCAM, soluble intercellular adhesion molecule.

## Discussion

The principal finding of our study—that coronary calcium scores are much lower in Ugandans compared with similarly aged persons in the USA, even after adjustment for cardiometabolic risk profile—is novel and challenges current assumptions about rising rates of ischaemic heart disease in SSA. These findings do not appear to be explained by population differences in race or smoking. In addition, we show an inverse association of nadir CD4+ T cell count with CAC among PLWH, and a positive association between biomarkers of inflammation and immune activation and calcified coronary plaque among Ugandans. Finally, the strength of the inflammation–CAC association appears to be strongest for PLWH compared with HIV-uninfected Ugandans. These findings must be interpreted within the context of the limited literature on subclinical coronary disease in SSA and a number of study limitations.

The Global Burden of Disease (GBD) Study 2017 estimates that the age-standardised prevalence of ischaemic heart disease is similar in the USA and Uganda (1776 vs 1293 per 100 000, USA vs Uganda).^15^ In SSA and other areas without primary data on ischaemic heart disease, the GBD Study uses statistical models to impute this disease burden. Moreover, the validity of these models depends on the quality of published data, which are notably lacking in much of SSA.^16^ In the absence of clinical outcomes data, we sought to use coronary calcium scoring as a reliably strong surrogate marker of ischaemic heart disease risk.^17 18^ Our findings of dramatically lower rates of CAC in Uganda suggest that rates of clinical ischaemic heart disease might in fact be lower than estimated by GBD; however, further population-based studies and better systems of CVD surveillance are needed to confirm this.

Few published studies have reported on CAC scores in SSA, and none to our knowledge have compared HIV-positive and HIV-uninfected persons. One study of younger, multiethnic South Africans with dialysis-dependent kidney disease (n=40 blacks and n=34 non-blacks; mean age 42 years, 14% diabetes) found significantly lower prevalence of CAC >0 among blacks (15%) compared with non-blacks (68%).^19^ Black African–Americans may have lower coronary calcification in US population-based studies such as the multi-ethnic Study of Atherosclerosis (MESA) and the coronary artery risk development in young adults study (CARDIA).^20^ For example, each SD increase in European ancestry does appear to be associated with a modest 8% higher CAC prevalence among African–Americans in MESA.^21^ Exclusion of non-black subjects from our final model, however, only modestly attenuated differences in CAC prevalence between the USA and Uganda. It is possible that higher European ancestry among African–Americans compared with Ugandans may be responsible for some degree of residual confounding; however, we feel this is unlikely to account for such dramatic differences in CAC prevalence in our study. Rather, we believe that *life-course* exposure to risk factors, diet, genetic predisposition and/or physical activity might be responsible for the regional differences we found. Since the process of urbanisation and incorporation of Western diet and lifestyle habits in Uganda is a relatively recent phenomenon, the current older adult Ugandan population is likely to have spent a considerable proportion of their lifetime with a limited risk factor exposure.^22^ These considerations should be examined in future studies of coronary atherosclerosis in SSA.

The presence and extent of CAC are an excellent surrogate marker of the total burden of coronary atherosclerosis and are predictive of future coronary events, across all racial and ethnic groups^17^ and across categories of 10-year Framingham risk.^18^ In particular, a CAC score of 0 is associated with extremely low rates of future ASCVD events among low to intermediate risk asymptomatic patients, and this effect is similar irrespective of race/ethnicity.^23^ However, future studies using contrast-enhanced CT coronary angiography could provide useful information about non-calcified plaque and high-risk plaque features to further characterise subclinical coronary atherosclerosis in this setting.

Chronic HIV infection, which is known to increase risk for CVD events in the USA and Europe,^2^ has been more strongly associated with non-calcified plaque^24^ and high-risk plaque features^25^ compared with calcified plaque. For example, in the Multicenter AIDS Cohort Study (MACS), HIV-positive status was associated with only modest and statistically borderline higher rates of CAC >0 in risk factor-adjusted models (prevalence ratio 1.12 (95% CI 1.08 to 1.35)), but the association with non-calcified plaque was somewhat stronger (prevalence ratio 1.25 (95% CI 1.10 to 1.43)). In unadjusted analyses, PLWH had higher odds of CAC >0 in our study, but similar to MACS this effect was attenuated after adjustment for risk factors. Our findings are consistent with a modest effect of HIV on subclinical coronary atherosclerosis in Uganda; however, we were not adequately powered given the low prevalence of CAC in both groups. Therefore, studies of non-calcified coronary plaque as well as clinical ASCVD event rates are needed.^2^

Among PLWH in our study, lower nadir CD4+ count was associated with higher odds of CAC >0. Prior studies among PLWH suggest that having a history of more severe immunosuppression (ie, low nadir CD4+ T cell count) is associated with more subclinical atherosclerosis, endothelial dysfunction and vascular stiffness.^24 26 27^ Lower nadir CD4+ T cell count is also associated with higher levels of chronic inflammation and immune activation even after viral suppression on ART.^28 29^

Chronic inflammation is a well-described risk factor for CVD. Among PLWH, markers of inflammation and immune activation have been variably associated with subclinical vascular disease, including CAC, non-calcified plaque and high-risk plaque features.^9–12 25^ No studies have previously evaluated these associations among HIV and HIV-uninfected persons in low-income and middle-income countries, such as Uganda. Interestingly, we found only modest differences in biomarker concentrations between HIV and HIV-infected subjects in our study. One marker of monocyte activation—sCD14—was higher among PLWH and among women in our cohort, mirroring findings from a south-west Uganda study.^30^ Another marker of monocyte activation—sCD163—was strongly associated with CAC >0 in our models after adjustment for traditional risk factors. Interestingly, this association was much stronger among PLWH than among HIV-uninfected Ugandans. Although plasma levels of oxLDL were lower among PLWH in Uganda, the strength of the association with CAC was greater. These findings support immune activation as an important CVD risk marker in SSA, particularly for PLWH. Future studies should further explore how novel drivers of these immune pathways such as chronic infections (eg, cytomegalovirus, malaria, helminths and latent tuberculosis) or exposure to environmental and indoor air pollution may affect cardiovascular risk in this context.

The relatively large sample size for a study from SSA, the inclusion of a US comparison group, and the inclusion of inflammation and immune activation markers are principal strengths of our study. In addition, our study included more women than is typical for studies of PLWH, which increases the generalisability of our findings. Limitations include the cross-sectional design and limited power to detect potentially important associations, including differences between subgroups. Our study subjects were recruited mostly from clinics rather than the community, which may affect the generalisability of our results to the general population. Additionally, differences in baseline risk factor profile between the Uganda and the US subjects allow the possibility of residual confounding.

## Conclusions

In conclusion, rates of coronary calcification were lower than anticipated in this study of PLWH and HIV-uninfected Ugandans compared with subjects from Cleveland, Ohio, USA. The extent to which HIV infection is associated with subclinical atherosclerosis and/or clinical ASCVD events in SSA requires larger community-based cohort studies. Chronic inflammation and immune activation may be important drivers of subclinical atherosclerosis and clinical ASCVD risk in SSA, particularly for PLWH.
