# Supplementary figures and images for: Coronary artery calcium, HIV and inflammation in Uganda compared with the USA
Source: Open Heart. 2019 May 22;6(1):e001046. doi: 10.1136/openhrt-2019-001046 (PMC6546194; doi:10.1136/openhrt-2019-001046)

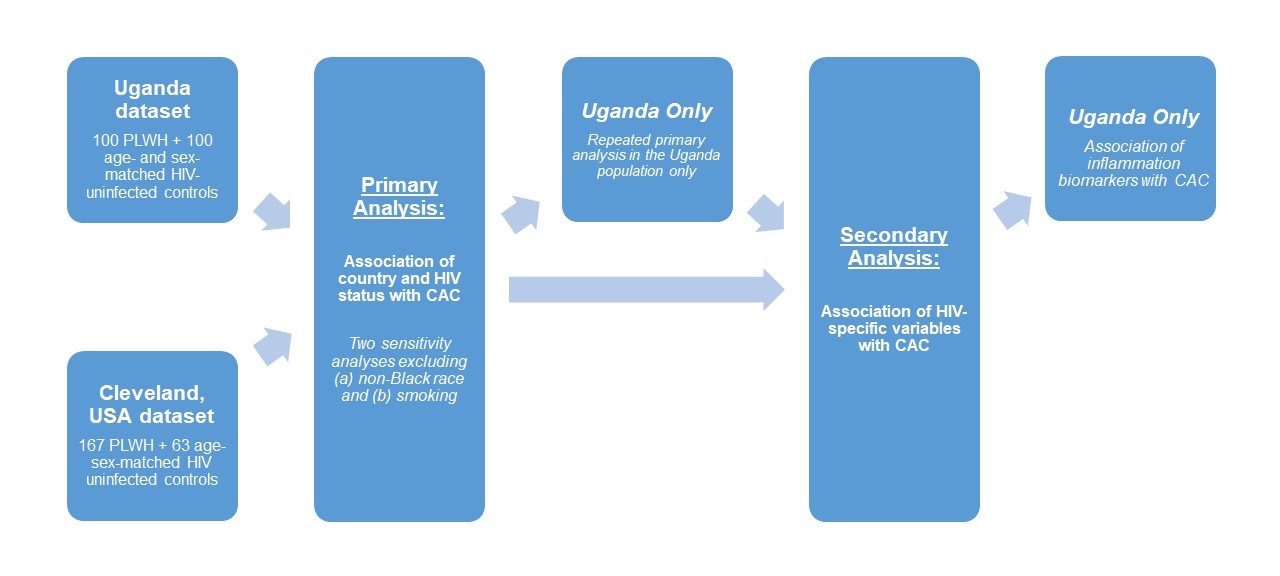

Supplement: Supplementary data [file openhrt-2019-001046supp001.jpg]
